# Comparison of Melatonin, Hypertonic Saline, and Hydroxyethyl Starch for Resuscitation of Secondary Intra-Abdominal Hypertension in an Animal Model

**DOI:** 10.1371/journal.pone.0161688

**Published:** 2016-08-25

**Authors:** Mingtao Chang, Hao Tang, Dong Liu, Yang Li, Lianyang Zhang

**Affiliations:** Trauma Center, State Key Laboratory of Trauma, Burns and Combined Injury, Institute of Surgery Research, Daping Hospital, Third Military Medical University, Chongqing, China; Max Delbruck Centrum fur Molekulare Medizin Berlin Buch, GERMANY

## Abstract

A variety of agents may have a beneficial effect in reducing injury-induced intestinal edema of fluid, but studies confirming the efficacy and mechanisms of these agents in secondary intra-abdominal hypertension (IAH) are lacking. This study was to compare the effectiveness of melatonin, 7.5% hypertonic saline (HS), and hydroxyethyl starch 130/0.4 (HES) on the resuscitation of secondary IAH in a rat model. Female SD rats were divided into: sham group, shock group, lactated Ringer solution (LR) group, melatonin group, HS group, and HES group. Except for the sham group, all rats underwent a combination of inducing portal hypertension, hemorrhaging to a MAP of 40 mmHg for 2 hr, and using an abdominal restraint device. The collected blood was reinfused and the rats were treated with LR (30ml/h), melatonin (50 mg/kg) + LR, HS (6 ml/kg) + LR, and HES (30 ml/kg) + LR, respectively. The shock group received no fluids. LR was continuously infused for 6hr. The intestinal permeability, immunofluorescence of tight junction proteins, transmission electron microscopy, level of inflammatory mediators (TNF-a, IL-1β, IL-6) and of biochemical markers of oxidative stress (malondialdehyde, myeloperoxidase activity, and glutathione peroxidase) were assessed. Expressions of the protein kinase B (Akt) and of tight junction proteins were detected by Western blot. Compared with LR, HS, and HES, melatonin was associated with less inflammatory and oxidative injury, less intestinal permeability and injury, and lower incidence of secondary IAH in this model. The salutary effect of melatonin in this model was associated with the upregulation of intestinal Akt phosphorylation.

## Introduction

Intra-abdominal pressure (IAP), the steady-state pressure concealed within the abdominal cavity, is approximately 5–7 mmHg in critically ill adults [1]. When a sustained or repeated pathological elevation in IAP≥12 mmHg, intra-abdominal hypertension (IAH) is present [1]. Secondary IAH refers to conditions that do not originate from the abdominopelvic region, and is commonly the result of such extra-abdominal injuries as sepsis, capillary leak, major burns, trauma, and disease requiring massive fluid resuscitation [2]. These states can present similar to systemic hypoperfusion and hypoxemia followed by subsequent resuscitation, which commonly result in a systemic ischemia/reperfusion injury with the hallmark of generalized edema or “third-spacing” of fluid. The same pathological process occurs in the intestine, resulting in secondary IAH. IAH has consistently been associated with significant organ failure, morbidity and mortality among critically ill adults [3–5]. Unfortunately, how to prevent this condition and improve patient outcomes with nonsurgical management at the beginning of treatment remains uncertain.

Fluid resuscitation is an essential therapy for hemorrhagic shock, and resuscitation aims to optimize tissue perfusion with adequate oxygen delivery, prevent or reverse end-organ failure, and minimize resuscitation-related complications during the resuscitation process. However, the amount of crystalloid fluids administered is a strong independent predictor of the development of secondary IAH [6]. Thus, a judicious fluid therapy must be used to minimize the expected sequelae. A variety of agents may have a beneficial effect in reducing injury-resuscitation-induced third-spacing of fluid, such as melatonin, colloids and hypertonic saline [7]. Although many experiments have been designed to prove the effectiveness of these agents in preventing intestinal injury, studies confirming their efficacy in secondary IAH are lacking [8–10]. The objective of this study was to examine the effects of melatonin, 7.5% hypertonic saline (HS), and hydroxyethyl starch 130/0.4 (HES) on secondary IAH in a pathophysiological rat model and the underlying mechanisms involved.

## Material and Methods

### Animals

All procedures were approved by the Institutional Animal Care Committee and were in accordance with the guidelines of the National Institute of Health regarding the care and use of animals. Female Sprague—Dawley (SD) rats (230–240 g) were provided by the Experimental Animal Center, Daping Hospital, Third Military Medical University, Chongqing, China. The rats were housed in temperature (22 ± 1°C) and humidity (60–65%)-controlled animal quarters with a 12-hr light/dark cycle. Animals were made to fast for 8 hr before the experiment but had free access to water ad libitum. Two hours before anesthesia, the rats were given a 2.0 ml solution of 100 mg lactulose and 50 mg mannitol by gastric tube feeding.

### Rat secondary IAH model

**Fig 1:**
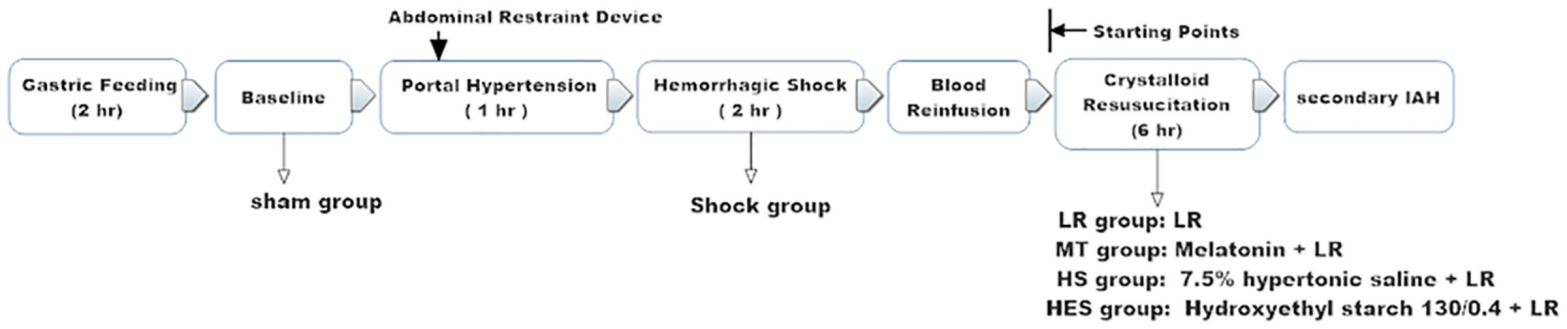
Schematic diagram of the experiment protocol. Rats were randomized into: sham group (sham, n = 8), no treatment was performed after anesthetization. Shock group (S,n = 8), no fluids were given after shock. Lactated Ringer solution (LR) group (LR, n = 8), after blood reinfusion, rats were resuscitated with LR (30 mL/h × 6hr). Melatonin group (MT, n = 8), after blood reinfusion, the rats were infused with melatonin (50mg/kg), then resuscitated with LR (30 mL/h × 6hr). Hypertonic saline group (HS, n = 8), after blood reinfusion, rats were infused with 7.5% hypertonic saline (6ml/kg), then resuscitated with LR (30 mL/h × 6hr). Hydroxyethyl starch group (HES, n = 8), after blood reinfusion, rats were infused with hydroxyethyl starch 130/0.4 (30ml/kg), then resuscitated with LR (30 mL/h × 6hr). The IVCP after blood reinfusion was set as the starting point, and the secondary IAH was determined by an elevation of 12.5 mmHg (170 mmH2O) of IVCP from the starting point.

**Fig 2:**
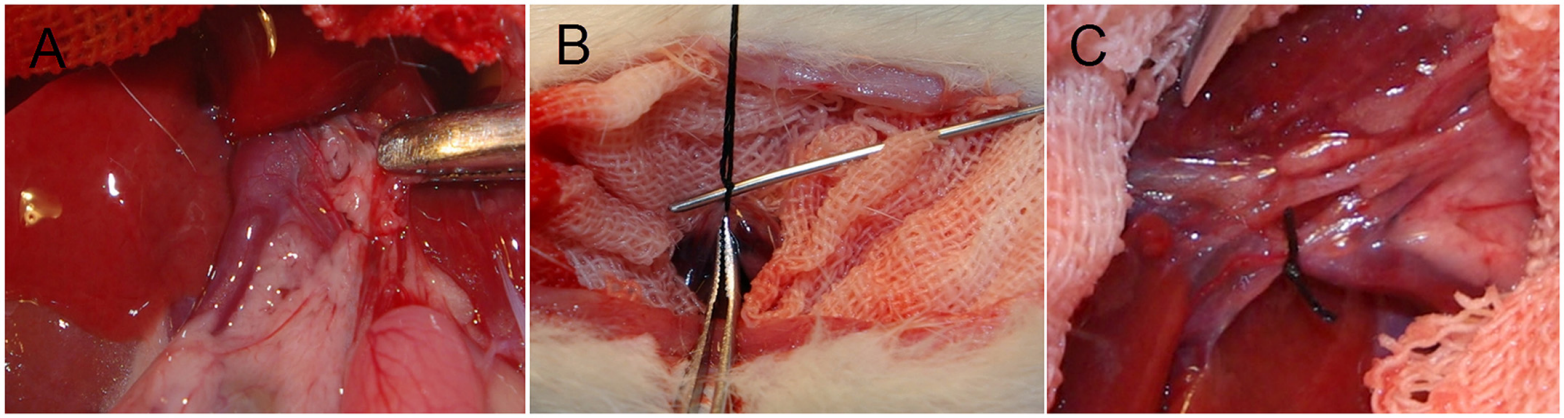
Induction of portal hypertension. (A) The portal vein was freed from the surrounding tissues. (B) A ligature (silk 4–0) was placed around a 21-gauge blunt-tipped needle lying alongside the portal vein. (C) Removal of the needle yielded a calibrated stenosis of the portal vein.

As previously described, a model of secondary IAH was used [11]. 2 hr after gastric feeding, the anesthesia was administered by an intraperitoneal injection of 30 mg/kg pentobarbital sodium (30 mg/mL). The anesthesia was maintained using pentobarbital sodium (15 mg/mL) through the right femoral vein, which was performed at a rate of 200 μl/hr by an infusion pump (B. Braun Perfusor Compact S Syrine infusion pump; B. Braun Melsungen AG, Melsungen, Germany). All animals were maintained in a fully anesthetized state from initial anesthesia to the time of sacrifice (from baseline stage to secondary IAH stage, as outlined in Fig 1). The rats were then immobilized in a supine position with forearm in abduction at 90° to the body, which was later extended. The right femoral vein was cannulated using polyethylene tubing (PE-50, Smiths-Medical, UK) for the infusion (ZNB-XB infusion pump; Beijing Kelly Med Co, Ltd, Beijing, China). The left femoral artery was cannulated for mean arterial pressure (MAP) monitoring (mercury manometer), and the left femoral vein was cannulated 3.5 cm in the inferior vena cava for inferior vena cava pressure (IVCP) monitoring (water manometer, zeroed at the level of the midaxillary line, 1 mm Hg = 13.6 mm H2O). All the cannulae contained normal saline with heparin (5 U/mL). Another catheter (outside diameter, 0.8 mm) was inserted into the bladder for the measurement of urine output and the collection of urine samples. To maintain body temperature at 37°C, the rats were placed on a warming plate. A laparotomy was performed, and the portal vein was freed from the surrounding tissues (Fig 2A). A ligature (silk 4–0) was placed around a 21-gauge blunt-tipped needle lying alongside the portal vein (Fig 2B). Subsequent removal of the needle yielded a calibrated stenosis of the portal vein, and the incision was closed with sutures (Fig 2C). Portal hypertension was induced 1 hr before shock.

**Fig 3:**
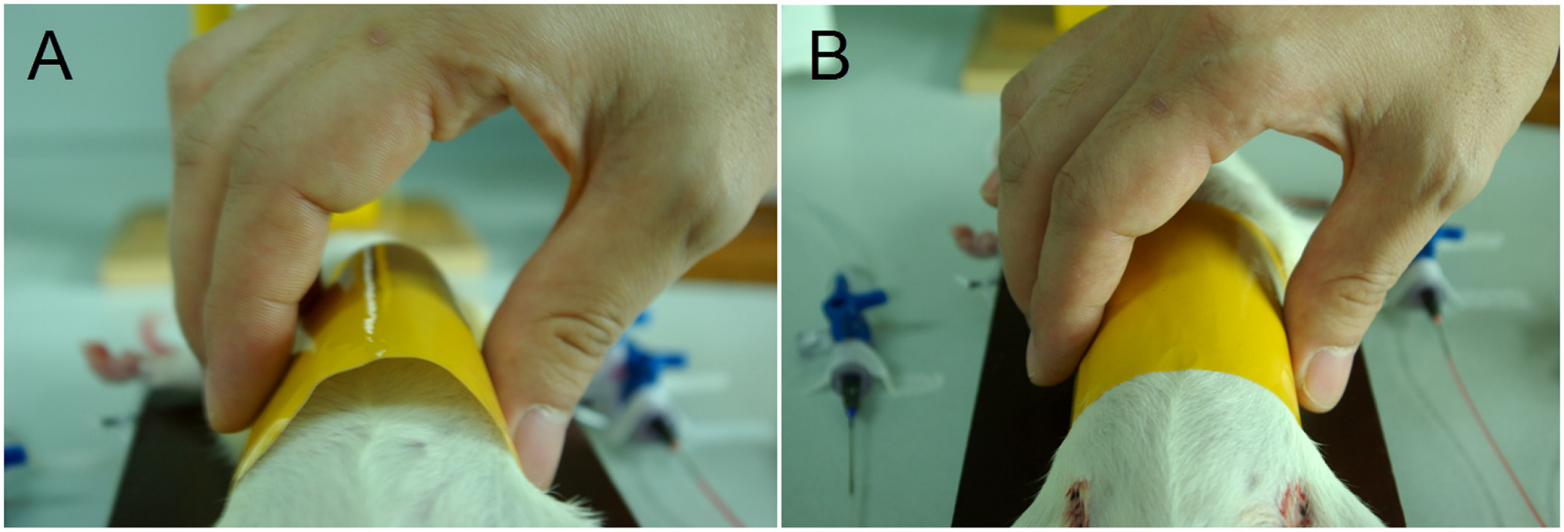
Use of the abdominal restraint device. (A) The abdominal constraint device was applied before resuscitation, ensuring a suitable abdominal compartment to contain hydropic organs and leaked fluid in a certain period of time.(B) After a period of resuscitation the device limits the expansion of the abdomen.

After the abdominal cavity was closed, a specially-designed device (circumference 15 cm, width 6 cm, soft, and non-ductile; Henkel, Shanghai, China) was loosely fitted to the rats' abdominal wall to mimic decreased abdominal wall compliance (Fig 3). This device did not compress the abdomen and affect the physiological parameters for circulation and ventilation. Hemorrhagic shock was induced by withdrawing arterial blood (0.5 mL/min) to a MAP of 39–42 mmHg, and MAP was maintained by blood withdrawal or infusion. The rats were left in shock for 2 hr, at the end of which the collected blood (anticoagulated with 100 U/mL heparin) was reinfused. After 15 min of stabilization, the infusion of lactated Ringer solution (LR) (30 mL/h) continued for 6hr.

### Experimental design

The rats were randomly divided into 6 groups (Fig 1). In the sham group (sham, n = 8), no treatment was performed after anesthetization. In the shock group (S, n = 8), rats were given no fluids after shock. In the LR group (LR, n = 8), after blood reinfusion, rats were resuscitated with LR (30 mL/h × 6hr). In the melatonin group (MT, n = 8), after blood reinfusion, rats were infused with melatonin (50mg/kg), then resuscitated with LR (30 mL/h × 6hr). In the hypertonic saline group (HS, n = 8), after blood reinfusion, rats were infused with 7.5% hypertonic saline (HS) (6ml/kg), then resuscitated with LR (30 mL/h × 6hr). In the hydroxyethyl starch group (HES, n = 8), after blood reinfusion, rats were infused with hydroxyethyl starch 130/0.4 (HES) (30ml/kg), then resuscitated with LR (30 mL/h × 6hr).

After blood reinfusion, the IVCPs of the rats were different. The IVCP after blood reinfusion was set as the starting point (when the IAP was 0 mmHg), and the secondary IAH was determined by an elevation of 12.5 mmHg (170 mmH2O) of IVCP from the starting point [1]. MAP, IVCP, infusion, and urine output were monitored. At the end of the experiment, the animals were sacrificed by an overdose of pentobarbital, and samples from intestine, blood and urine were harvested. Rats in the shock group were euthanized before blood reinfusion. Blood samples were immediately centrifuged at 3000 rpm for 20 mins, and the resulting supernatants and intestinal samples were frozen in liquid nitrogen for molecular, biological and biochemical experiments.

### Intestinal permeability measurements

Intestinal permeability was quantified using urinary excretion ratio of the lactulose/mannitol as previously described [12]. Two hours before anesthesia, the rats were given 2.0 ml of a test solution containing 100 mg lactulose (Sigma, St Louis, USA) and 50 mg mannitol (Sigma, St Louis, USA) by gastric tube feeding. All urine was recorded and collected by urethral catheterization throughout the experiment and stored at -80°C for later analysis. The urine of all eight rats of each group was quantitatively analyzed by high-performance liquid chromatography (HPLC, Waters Company, USA). Results were expressed as a ratio of percentage of the administered dose of the two molecules excreted.

### Enzyme-linked immunosorbent assay (ELISA)

The levels of inflammatory mediators and the biochemical markers of oxidative stress were quantified using specific ELISA kits for all eight rats of each group according to the manufacturers’ instructions (tumor necrosis factor alpha(TNF-a), interleukin-1β(IL-1β), interleukin-6 (IL-6), myeloperoxidase (MPO) activity, malondialdehyde (MDA), glutathione peroxidase (GSH-Px) from Merck Millipore, MA, USA; Diamine oxidase (DAO) from Nanjing Jianchen Bioengineering Institute, Nanjing, China).

### Transmission electron microscopy (TEM)

Six rats of each group were selected at random for transmission electron microscopy. After collection, intestinal samples were fixed with 2.5% glutaraldehyde and processed for TEM following standard procedures. Ultra-thin sections were mounted on copper grids, contrasted with standard uranyle acetate and lead citrate double staining, and observed in a HITACHI-7500 (Tokyo, Japan) transmission electron microscope at 80 kV at a magnification of 40,000. To evaluate changes in tight junction ultrastructure, only sections with longitudinally oriented microvilli were examined in each sample. Examinations were performed independently by two investigators in a blinded manner.

### Immunofluorescence

Six rats of each group were selected at random for immunofluorescence. After collection, tissues of were fixed in 4% paraformaldehyde and embedded in paraffin. Blocks were sectioned (5 μm), mounted on slides, then deparaffinized and rehydrated by successive incubations in xylene, 100% ethanol, 95% ethanol, 80% ethanol, 70% ethanol, and phosphate buffer saline (PBS). Custom anti-occludin antibodies (Abcam, Cambridge, MA, USA) were applied overnight at 4°C, followed by an Alexa 488–conjugated goat anti-rabbit IgG antibody for 60 min (Abcam, Cambridge, MA, USA). The slides were washed extensively and stained with DAPI (4’6’-diamidino-2-phenylindole) (Abcam, Cambridge, MA, USA). Images were obtained (Zeiss LSM 780 laser confocal microscopy) at excitation wavelengths of 390 nm and 500 nm; emission was detected at 440 and 580 nm. All the stainings were performed in duplicate in non-serial distant sections, and analyzed in a double-blind manner by two different experienced investigators.

### Western blot analysis

Western blot analysis was performed in the standard fashion. Intestinal tissues from each rat were homogenized in a buffer (10 mM Tris HCl, 250 mM sucrose, 2 mM PMSF, protease inhibitor cocktail; pH 7.4), and centrifuged at 15,000g for 30 min at 4°C. The upper fluffy layer of the pellet was resuspended in the homogenization buffer and was considered to be the membrane fraction, while the supernatant was considered to be the cytosolic fraction. The samples were quickly frozen and stored at -80°Cuntil use. Equal amounts of proteins (50μg) were resolved in 10% SDS-PAGE and transferred onto nitrocellulose membranes. The membranes were blocked with 5% nonfat dry milk in Tris-buffered saline (TBS) with 0.05% Tween 20, for 90 min at room temperature. The membranes were incubated with anti-phospho-Akt (1:2000; Cell Signaling Technology, Danvers, MA, USA), anti-Akt (1:1000; Cell Signaling Technology, Danvers, MA, USA), anti- zonula occludens-1(ZO-1) (1:50; Abcam, Cambridge, MA, USA), and anti-occludin (1:100; Abcam, Cambridge, MA, USA) antibodies overnight at 4°C. The membrane-bound antibodies were visualized using IRDye 800 infrared-labeled donkey anti-rabbit secondary antibodies (1:15 000;Li-Cor Bioscience, Lincoln, NE,USA)and the Odyssey Infrared Imaging System (Li-Cor Bioscience, Bad Homburg, Germany). The densities of the bands were quantified by densitometry using the Quantity-One software (Bio-Rad, Hercules, CA) and normalized with actin (Sigma, St Louis, USA).

### Statistical analysis

All data were expressed as the mean ± standard error of the means (S.E.M.). The statistical significance between groups was assessed with one-way analysis of variance followed by the Tukey's post hoc test. Values of p<0.05 were regarded as significant. All analyses were performed using SPSS 16.0 software (SPSS Inc, Chicago, IL).

## Results

### Melatonin reduces the incidence of secondary IAH compared with other agents

As shown in Table 1, the average amount of melatonin given in MT group was (11.71±0.12) mg. The average amount of 7.5% hypertonic saline given in HS group was (1.41±0.02) ml, and the average amount of hydroxyethyl starch 130/0.4 given in HES group was (7.03±0.06) ml.

**Table 1:** The type and amount of fluids given in each group.

Group	Type of fluids	Amount of fluids
sham	No fluids	No fluids
S	No fluids	No fluids
LR	LR (30ml/h × 6h)	LR:180ml
MT	melatonin (50mg/kg) + LR (30ml/h × 6h)	melatonin:11.71 ± 0.12 mg; LR:180ml
HS	hypertonic saline (6ml/kg) + LR (30ml/h × 6h)	hypertonic saline:1.41± 0.02 ml; LR:180ml
HES	hydroxyethyl starch (30ml/kg) + LR (30ml/h × 6h)	hydroxyethyl starch:7.03 ± 0.06 ml; LR:180ml

The rats were randomly divided into 6 groups. In the sham group and the Shock group (S group, n = 8), rats were given no fluids. Animals subjected to resuscitation were treated with lactated Ringer solution (LR group,30 mL/h × 6hr), melatonin plus LR (MT group, 50mg/kg), 7.5% hypertonic saline plus LR (HS group, 6ml/kg), or hydroxyethyl starch 130/0.4 plus LR(HES group, 30ml/kg). Data of the amount of fluids are presented as means ± S.E.M., n = 8 in each group.

**Fig 4:**
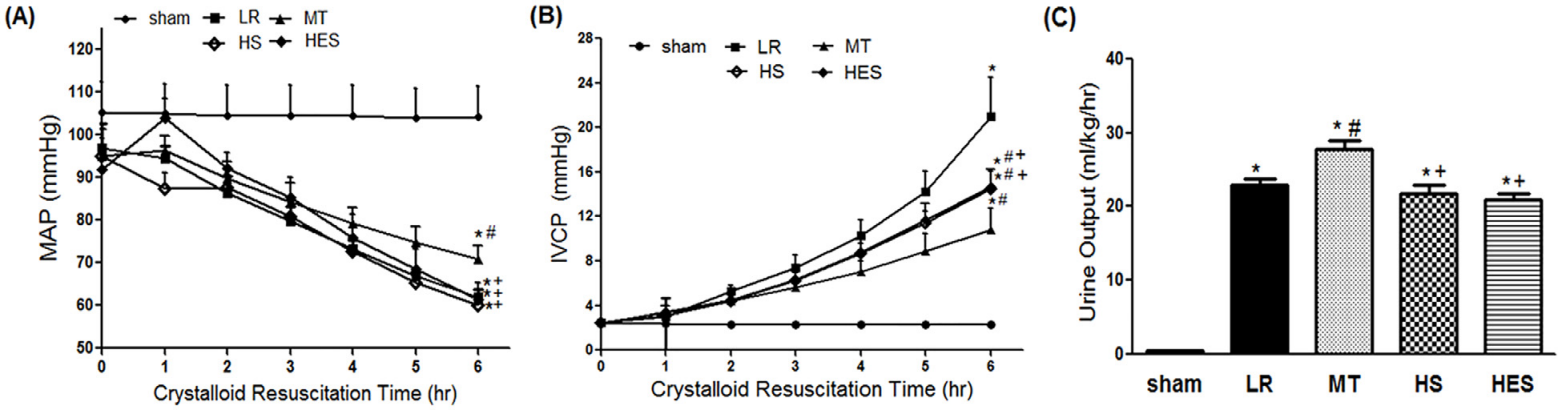
Changes in the mean arterial pressure (MAP) and inferior vena cava pressure (IVCP) during the crystalloid resuscitation time and comparisons of urine output between groups. (A) Changes in the MAP of groups during the crystalloid resuscitation time. (B) Changes in the IVCP of groups during the crystalloid resuscitation time. (C) Comparison of urine output during the crystalloid resuscitation time. Data are presented as means ± S.E.M., n = 8 in each group. * *P* < 0.05 in comparison with the sham group, ^*#*^ *P* < 0.05 in comparison with the LR group, ^+^ *P* < 0.05 in comparison with the MT group.

As shown in Fig 4A, resuscitation resulted in a decrease in MAP in the LR group, MT group, and HS group. But in the HES group, MAP increased to nearly normal in the first hour, then decreased in the following 5hr. In the MT group, MAP had a progressive decline, and after 6hr resuscitation MAP was higher in the MT group (70.9±3.2 mmHg) compared with the LR group(61.9±1.9 mmHg, p<0.001), HS group(60.0±1.9 mmHg, p<0.001) and HES group (61.3±4.1 mmHg, p<0.001). After 6hr resuscitation, there was no significant difference between the LR group, HS group and HES group(p>0.05).

As shown in Fig 4B, resuscitation resulted in an elevation in IVCP in the LR group, MT group, HS group and HES group. In the LR group, all rats developed secondary IAH from the starting point (20.99±3.50 mmHg). In the HS group, two rats developed secondary IAH from the starting point (final IVCP:15.39mmHg, 13.38 mmHg, respectively). In the HES group, three rats developed secondary IAH from the starting point (final IVCP: 13.38mmHg, 13.61 mmHg, 14.27 mmHg, respectively). But in the MT group, no secondary IAH model emerged (10.84 ± 1.89 mmHg). After 6hr resuscitation, IVCP in the MT group was lower compared with the LR group (20.99±3.50 mmHg, p<0.001), HS group(14.51±1.55 mmHg, p<0.05) and HES group (14.63±1.61 mmHg, p<0.05), and IVCP in the LR group was higher compared with the other groups(p<0.001). There was no significant difference between the HS group and HES group (p>0.05).

As shown in Fig 4C, urine output increased significantly in the MT group compared with the LR group (27.80 ± 3.38 mL/kg/h versus 22.88 ± 2.46 mL/kg/h, p<0.01). In the HS group and HES group, urine output decreased, and no significant difference was observed compared with the LR group(p>0.05). But there were significant differences between the MT group and the HS (21.79 ± 3.02 mL/kg/h, p<0.01) and HES groups (20.85 ± 2.53 mL/kg/h, p<0.001).

### Melatonin reduces the increase of intestinal permeability compared with other agents

**Fig 5:**
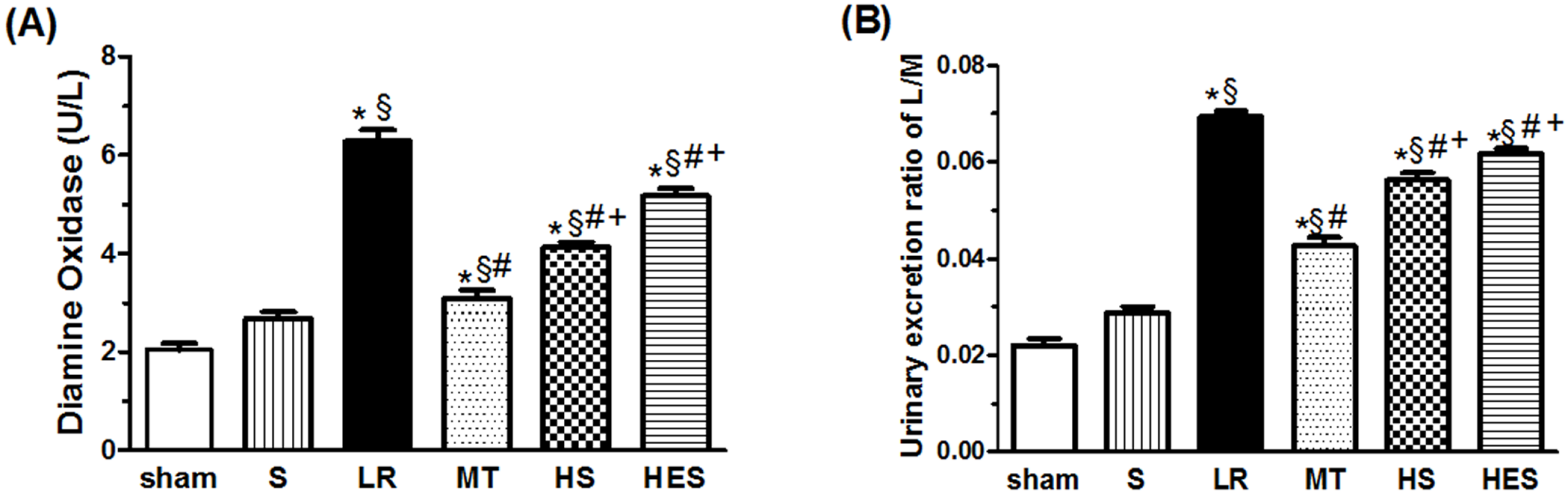
Levels of intestinal diamine oxidase (DAO) and urinary excretion ratio of the lactulose/mannitol (L/M). (A) Comparison of intestinal DAO between groups. (B) Comparison of L/M between groups. Data are presented as means ± S.E.M., n = 8 in each group. Data are presented as means ± S.E.M., n = 8 in each group. * *P* < 0.05 in comparison with the sham group, ^*§*^ *P* < 0.05 in comparison with the S group, ^#^ *P* < 0.05 in comparison with the LR group, ^+^ *P* < 0.05 in comparison with the MT group.

As shown in Fig 5A, hemorrhagic shock (S group) resulted in an increase in DAO compared with sham group (2.67±0.39 versus 2.04±0.38 U/L, p<0.05). But resuscitation with LR resulted in a more significant increase(p<0.001). The treatment with melatonin reduced the level of DAO compared with the LR group (3. 09±0.49 versus 6.28±0.66 U/L, p<0.001), and the treatment with HS (4.13±0.29, p<0.001) and HES (5.12±0.41, p<0.001) also reduced the level of DAO compared with the LR group. Single administration of melatonin, HS, and HES with LR all decreased the LR resuscitation-induced increase in DAO, but the reduction in the MT group was more than the HS(p<0.01) or HES group(p<0.001).

As shown in Fig 5B, resuscitation with LR resulted in an increase in L/M compared with the S group (0.0693±0.0032 versus 0.0287±0.0038, p<0.001). The treatment with melatonin reduced L/M compared with the LR group (0.0427±0.0049 versus 0.0693±0.0032, p<0.001). The administration of HS(p<0.001) or HES(p<0.01) with LR also decreased the LR-induced increase in L/M, but the reduction in HS group(p<0.001) or HES group(p<0.001) was less than in the MT group.

### Melatonin ameliorates intestinal injury compared with other agents

**Fig 6:**
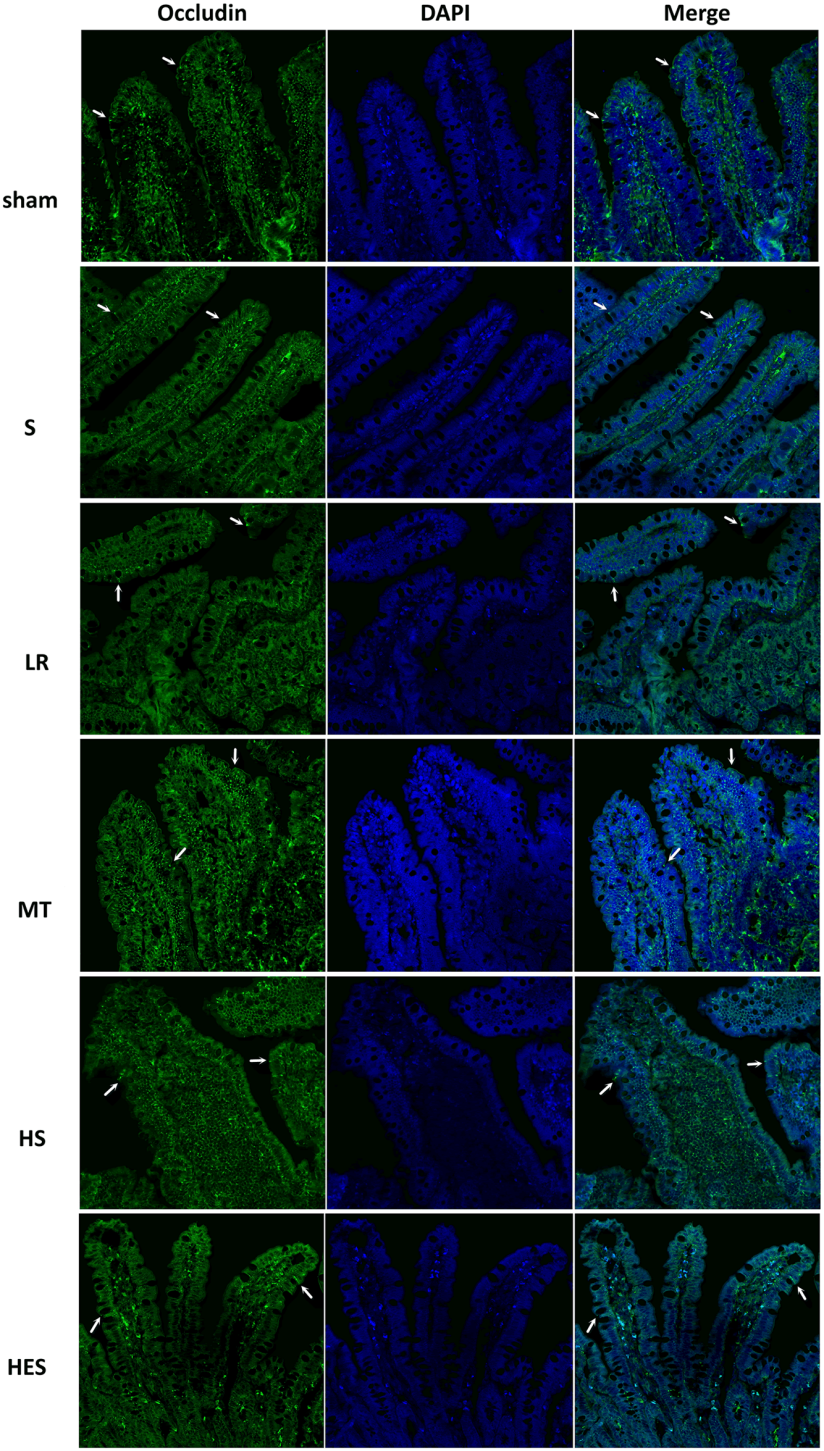
Representative micrographs of distribution of occludin between groups. The tight junctions protein Occludin (green) and nuclei (blue) images were obtained by immunofluorescence analysis of tissue sections of rats jejunums (200X). Arrows indicate areas of staining. Sections from at least 4 mice were examined for each condition.

As shown in Fig 6, occludin appeared as continuous bands along the epithelial sheet from the crypt to the villous tip in the sham group. In the S group, occludin appeared less frequently. In the LR group, the distribution of occludin was completely disrupted. Treatment of rats with melatonin could largely attenuate LR resuscitation-induced redistribution of occludin, and the distribution was similar to that in S rats. The administration of HS or HES with LR could also ameliorate the destruction, but the intensity of staining was less than that observed in Vehicle and MT mice.

**Fig 7:**
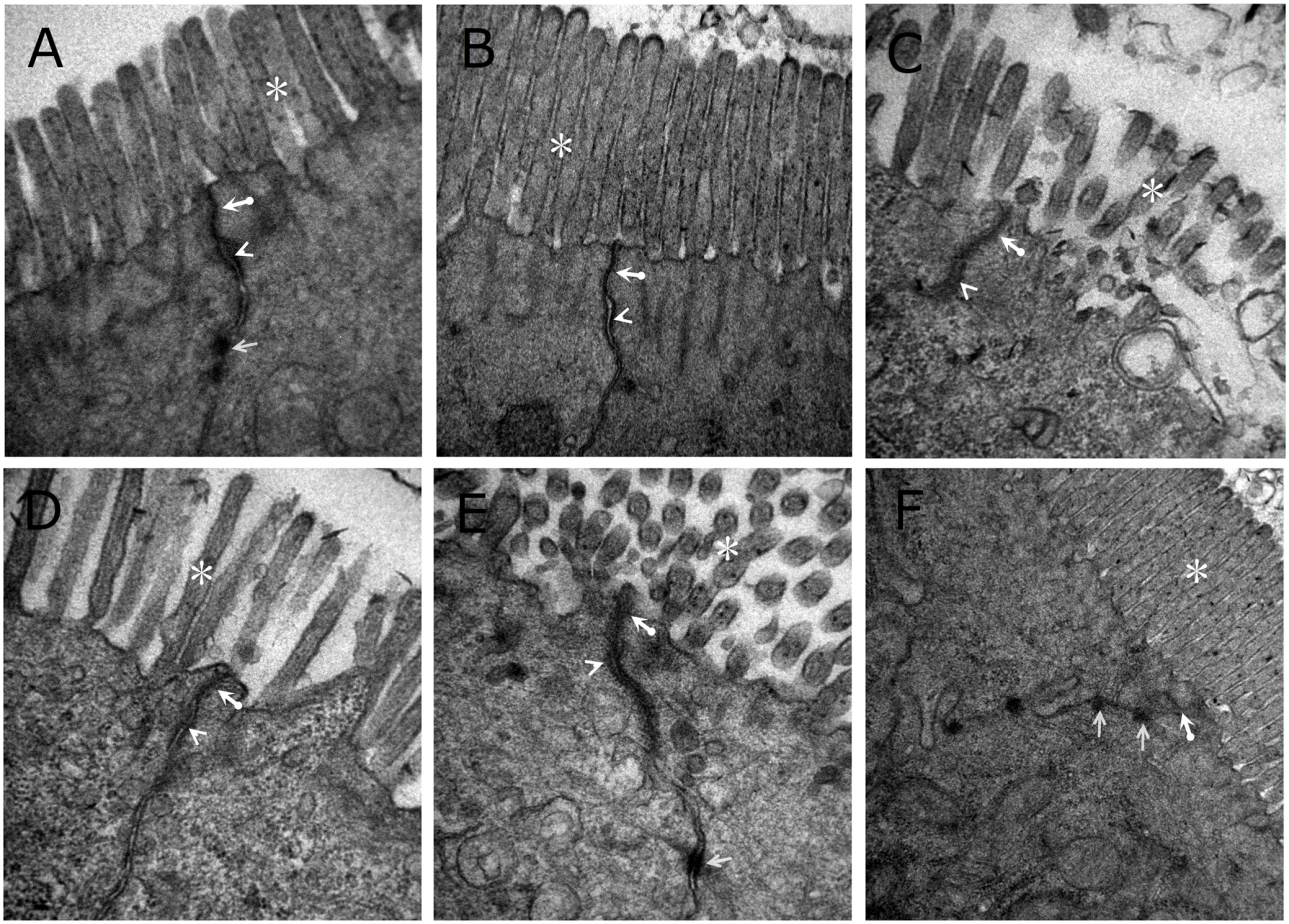
Representative photographs of transmission electron microscope of tight junctions in intestine (40 000X) from each group. (A) sham group. (B) S group. (C) LR group. (D) MT group. (E) HS group. (F) HES group. Thick arrows: tight junctions. Arrow heads: adherence junctions. Arrows: desmosome. Asterisks: microvilli.

As shown in Fig 7, in the sham group and S group, intact tight junctions structures were observed, and the microvilli was regularly aligned in the intestinal epithelium. In the LR group, the architecture of tight junctions was disrupted, and the electron-dense materials were less. Moreover, the amount of microvilli decreased and the arrangement was irregular in the LR group. The group subjected to treatment with melatonin protected the cytoarchitecture of the intestinal barrier and reduced the loss of microvilli. In the HS group, a less significant protective effect on tight junctions was presented than in the MT or HES groups. The group subjected to treatment with HES significantly protected the loss of microvilli, but the electron-dense materials were less.

### Melatonin attenuates oxidation and inflammation compared with other agents

As shown in Table 2, there was an increase in the levels of the intestinal oxidative injury mediators MDA(p<0.05) and MPO(p<0.01) in the shock group compared with the sham group. There was a decrease in GSH-Px in the shock group compared with the sham group(p<0.001). LR resuscitation after hemorrhage worsened these markers (p< 0.001 for MDA, MPO and GSH-Px). Administration of melatonin, HS, and HES with LR resuscitation all improved in the above-mentioned markers, but significant differences were observed in MT group compared with HS group (p< 0.001 for MDA, MPO and GSH-Px) and HES group (p< 0.05 for MDA, MPO and GSH-Px).

**Table 2:** Intestinal inflammatory and oxidative injury mediators in each group.

Group	MDA	MPO	GSH-Px	TNF-α	IL-1β	IL-6
sham	0.57 ± 0.10	1.35 ± 0.28	71.42 ± 7.52	25.56 ± 3.25	53.43 ± 8.28	169.59 ± 20.91
S	0.86 ± 0.13 *	2.27 ± 0.39 *	55.22 ± 6.71 *	37.98 ± 6.37	102.86 ± 24.26 *	298.86 ± 40.56 *
LR	2.13 ± 0.31 *^.^^§^	6.18 ± 0.56 *^.^^§^	33.90 ± 5.99 *^.^^§^	141.00 ± 26.52 *^.^^§^	375.70 ± 28.46 *^.^^§^	716.74 ± 51.55 *^.^^§^
MT	1.15 ± 0.28 *^.^^#^	3.69 ± 0.49 *^.^^§^^.^^#^	53.82 ± 6.59 *^.^^#^	47.44 ± 4.07 *^.^^§^^.^^#^	203.90 ± 18.83 *^.^^§^^.^^#^	493.59 ± 26.15 *^.^^§^^.^^#^
HS	1.72 ± 0.32 *^.^^§^^.^^#^^.^^+^	4.96 ± 0.39 *^.^^§^^.^^#^^.^^+^	35.76 ± 3.98 *^.^^§^^.^^#^^.^^+^	75.93 ± 4.99 *^.^^§^^.^^#^^.^^+^	310.70 ± 21.49 *^.^^§^^.^^#^^.^^+^	551.25 ± 31.98 *^.^^§^^.^^#^^.^^+^
HES	1.33 ± 0.28 *^.^^§^^.^^#^^.^^+^	4.35 ± 0.41 *^.^^§^^.^^#^^.^^+^	45.57 ± 4.88 *^.^^§^^.^^#^^.^^+^	60.11 ± 4.59 *^.^^§^^.^^#^^.^^+^	283.90 ± 27.10 *^.^^§^^.^^#^^.^^+^	501.66 ± 41.66 *^.^^§^^.^^#^

Animals subjected to resuscitation were treated with lactated Ringer solution (LR group,30 mL/h × 6hr), melatonin plus LR (MT group, 50mg/kg), 7.5% hypertonic saline plus LR (HS group, 6ml/kg), or hydroxyethyl starch 130/0.4 plus LR(HES group, 30ml/kg). MDA, malondialdehyde (nmol/mg prot); MPO, myeloperoxidase (U/g prot); GSH-Px, glutathione peroxidase (U/mg prot); TNF-a, tumor necrosis factor alpha (pg/ml); IL-1β, interleukin-1β (pg/ml); IL-6, interleukin-6 (pg/ml).

Data are presented as means ± S.E.M., n = 8 in each group.

* *P* < 0.05 in comparison with the sham group,

^*§*^ *P* < 0.05 in comparison with the S group,

^#^ *P* < 0.05 in comparison with the LR group,

^+^ *P* < 0.05 in comparison with the MT group.

As shown in Table 2, there was a marked increase in the levels of the intestinal inflammatory markers, IL-1β(p<0.01) and IL-6(p<0.001) in the shock group compared with the sham group. But in TNF-a,no significant difference was observed between the sham group and S group. LR resuscitation after hemorrhage worsened in the above-mentioned markers (p< 0.001 for TNF-a, IL-1β and IL-6). Administration of melatonin, HS or HES with LR resuscitation all improved in the above-mentioned markers, but significant differences were observed in the MT group compared with the HS group (p< 0.001 for TNF-a and IL-1β; p< 0.01 for IL-6) and HES group (p< 0.001 for TNF-a and IL-1β). There were no significant differences in the IL-6 between the MT group and HES group (p > 0.05).

### Melatonin improves the reduction of tight junction proteins in intestine possible via modulation of Akt

**Fig 8:**
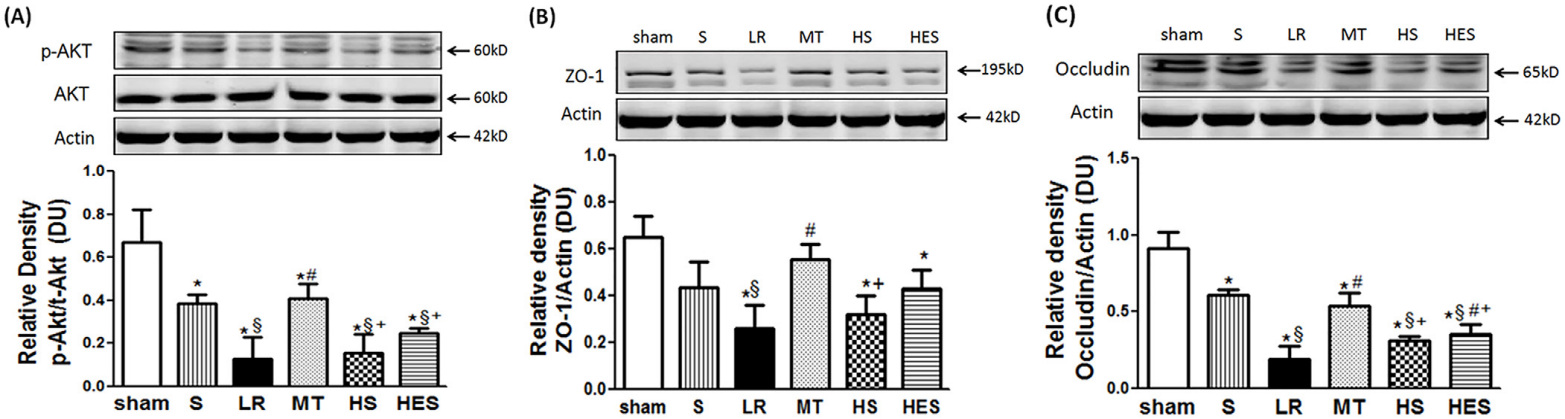
Expression of total and phosphorylated protein kinase B (p-Akt) and tight junction proteins in intestine between groups. The intestinal sample extracts were immunoblotted for the expression of phosphorylated (p)-Akt, total (t)-Akt, ZO-1 and occludin. Representative images of the Western blots are shown (A). Expression of p-Akt and t-Akt among groups (B); Expression of ZO-1 among groups; (C). Expression of occludin among groups. Data are presented as means ± S.E.M., n = 8 in each group. Data are presented as means ± S.E.M., n = 8 in each group. * *P* < 0.05 in comparison with the sham group, ^*§*^ *P* < 0.05 in comparison with the S group, ^#^ *P* < 0.05 in comparison with the LR group, ^+^ *P* < 0.05 in comparison with the MT group.

As shown in Fig 8A, hemorrhage (shock group) resulted in a decrease in Akt phosphorylation (p-Akt) compared with the sham group (p<0.05). But resuscitation with LR resulted in a significant decrease in p-Akt levels compared with the sham group (p<0.001). Only in MT(p<0.01) group, intestinal levels of p-Akt were significantly increased compared with LR group. No statistical difference was observed in the HS group and HES group compared with LR group (p>0.05). The MT group had a significant increase compared with the HS group(p<0.01) and HES group(p<0.05). No difference in total AKT protein expression was observed among groups (p>0.05).

As shown in Fig 8B, hemorrhage (shock group) resulted in a decrease in ZO-1 compared with the sham group, but no statistical difference was observed (p>0.05). ZO-1 expression decreased significantly after LR resuscitation compared with the shock group (p<0.01). Intestinal levels of ZO-1 significantly increased in melatonin-treated rats in comparison with LR group animals (p<0.01). No statistical differences were observed in the HS group and HES group in comparison with LR group (p>0.05). The MT group had a significant increase compared with the HS group(p<0.05).

As shown in Fig 8C, hemorrhage (shock group) resulted in a decrease in occludin compared with the sham group (p<0.01). Occludin expression decreased more significantly after LR resuscitation compared with the shock group (p<0.001). In MT group (p<0.001) and HES group (p<0.05), intestinal levels of occludin were significantly increased compared with LR group. No statistical difference was observed in the HS group and LR group (p>0.05). But the MT group had a significant increase compared with the HS group(p<0.05) and HES group(p<0.05).

## Discussion

This is the first study to compare the effects of melatonin, HS, and HES in an animal model of secondary IAH. Compared with LR, HS and HES, superior effects of melatonin are observed on reducing inflammatory responses and oxidative injury, attenuating intestinal injury and reducing the incidence of secondary IAH. And the protective effect of melatonin may be associated with upregulation of Akt phosphorylation.

Resuscitation and the development of secondary IAH are closely associated and frequently overlapping critical care topics [3, 13]. In past decades, conventional fluid resuscitation with LR has been challenged by numerous studies documenting its side effects, such as inflammatory response, capillary leak, and organ edema [14–16]. Hypertonic saline (7.5% NaCl) has been shown to be effective in smaller volumes when used as a resuscitative fluid after hemorrhagic shock [17, 18]. HS can suppress the initial inflammatory response after traumatic shock [19], eliminate intestinal tissue edema formation and improve intestinal transit [17]. However, HS, as a crystalloid solution, remains only 30–60 minutes within the intravascular space for a single use. And the effect of HS in continuous crystalloid resuscitation, which is widely accepted as the standard therapy for shock resuscitation by clinicians, remains unclear. We showed that the following large volume of LR infusion diluted HS and weakened HS treatment. And repetitious application of HS instead of single use during fluid therapy necessitates study. HES are generally considered to be more potent plasma volume expanders, with rapid and longer-lasting circulatory stabilization than crystalloids. And the macromolecules of HES could act as a sealant of endothelial gaps, preventing fluid loss and reducing stasis and interstitial edema [20, 21]. Chen and colleagues reported that fluid resuscitation with HES 130/0.4 after hemorrhagic shock was associated with lesser oxidative stress and a less severe inflammatory response in the intestine [9]. By contrast, in the current study, increased inflammatory and oxidative injury was evident after fluid resuscitation using HES. The differences are likely related to two aspects. First, hemorrhage time was 2hr in our model. The longer the period of ischemia is, the more severe cellular damage is present. Second, instead of synthetic colloid, HES plus 6hr LR resuscitation was used in the current study. This overzealous fluid resuscitation did not improve outcome, but facilitated inflammatory injury and the development of edema.

Melatonin, the main product of the pineal gland, of which the therapeutic effects against ischemia/reperfusion injuries in various organs have been extensively documented [22–24]. Without expectation, hypertonic crystalloid and colloid-based resuscitation has long be studied and recommended to improve the outcome after shock [25, 26]. And these two fluids are almost used in single small doses. In other words, they are more than just fluids, they are fluid drugs. By contrast, very few studies compare the therapeutic potential of melatonin with these two fluids drugs in the ischemia/reperfusion setting. In previous study, melatonin has been reported to be protective in reducing the intestinal permeability [8, 27]. However, these studies focused on the therapeutic value of melatonin in alcoholic liver disease, celiac disease, diabetes mellitus, and intestinal inflammation, which could result in intestinal barrier dysfunction. Since increased intestinal permeability is one of the key factors of secondary IAH, salutary effect of melatonin in the development of ischemia/reperfusion-induced secondary IAH should not be ignored. In the current study, as compared with HS and HES animals, superior effects of melatonin were found in inhibition of inflammation and reduction of intestinal permeability.

Some reports suggest that AKT pathway is involved in regulation of the immune responses [28]. In the intestinal ischemia/reperfusion models, epigallocatechin-3-gallate performed its protective role by enhancing the activation of Akt signaling pathway to suppress inflammatory response [29]. Another study also showed that erythropoietin ameliorates the acute intestinal injury by activating PI3K/Akt signaling to suppress NF-κB-mediating inflammation [30]. In our study, melatonin also activated Akt signaling to suppress inflammatory responses and ameliorated intestinal injury. But the level of phosphorylated Akt in agents group was lower compared with sham group, whereas these studies do not. Ischemic time and treatment of reperfusion are all relevant to the severity of ischemia/reperfusion injury. The published data manifested variations in terms of experimental conditions such as duration and degree of ischemia, treatment of reperfusion, and so on. Now that by regulating different signal pathways cells possess self-healing and self-protection in ischemia/reperfusion, over resuscitation after ischemia may lead to the decrease of this capability.

To study IAH, several animal models were chosen. IAP was artificially elevated by insufflation of gas [31, 32], instillation of fluids [33], and insertion of balloons or solid materials [34, 35]. However, none of these models could reproduce the pathophysiological processes triggered by capillary leak and fluid accumulation. As Schachtrupp and coworkers [36] have emphasized, the “next step in animal research would be the development of a ‘pathologic’ model in which hemorrhage (sic) or systemic inflammation together with resuscitation lead to abdominal fluid accumulation and increased intra-abdominal pressure.” Therefore, we chose a newly-created physiologically relevant rodent model of secondary IAH [11], that included the similar pathophysiological processes of hemorrhage, excessive resuscitation and damage control surgery, and that was suitable for studying the secondary IAH-induced injury and the effects of therapeutic interventions.

In conclusion, the results of the present study indicate that compared with HS and HES, melatonin may represent a new promising approach for the prevention of secondary IAH. The protective effects of melatonin seem to be due to downregulation of oxidative stress and inflammation and protection of intestinal barrier function injury via upregulation of Akt phosphorylation. We hope that our study will provide the theoretical and experimental basis for future preventative treatments of secondary IAH.
